# Colorimetric Determination of the Activity of Starch-Debranching Enzyme via Modified Tollens’ Reaction

**DOI:** 10.3390/nano9091291

**Published:** 2019-09-10

**Authors:** Ke Luo, Nack-geun Kim, Sang-Mook You, Young-Rok Kim

**Affiliations:** Graduate School of Biotechnology & Department of Food Science and Biotechnology, College of Life Sciences, Kyung Hee University, Yongin 17104, Korea

**Keywords:** silver nanoparticles, pullulanase, starch-debranching, Tollens’ reaction, colorimetric assay, enzyme activity

## Abstract

Nelson–Somogyi and 3,5-dinitrosalicylic acid (DNS) assays are the classical analytical methods for the determination of activity of starch-debranching enzymes, however, they have a narrow detection range and do not adapt to the quantitative measurement of linear polysaccharides. Herein, we developed a simple and accurate colorimetric assay for determining the activity of starch-debranching pullulanase through the modified Tollens’ reaction in combination with UV irradiation. Silver nanoparticles (AgNPs) were formed by reducing aldehyde groups in short-chain glucans (SCGs) generated by debranching of waxy maize starch using pullulanase through the modified Tollens’ reaction. In addition to providing a reducing moiety to the Tollens’ reaction, the debranching product, SCGs, also enhanced the colloidal stability of synthesized AgNPs, of which the amplitude of its surface plasmon resonance (SPR) absorbance peak was proportional to the concentration of SCGs ranging from 0.01–10 mg/mL. The detection limit of this system was 0.01 mg/mL, which was found to be 100 times higher than that of the conventional DNS assay. The purification of SCGs by recrystallization and gelatinization improved the selectivity of this colorimetric assay for debranching products, which provides a simple and accurate means of monitoring the debranching process and characterizing the activity of starch-debranching enzymes.

## 1. Introduction

Amylopectin, a highly branched polymer of α-glucose, is a major constituent of plant starch granules. Amylopectin has a high frequency of branch point at around 5–6% of the molecules, which results in a very complex molecular structure. Thus, the enzymes that can directly cleave the α(1,6) linkages are essential for plant metabolism. Such enzymes are termed starch-debranching enzymes (DBEs). DBEs are classified into two groups, namely pullulanase-type DBEs and isoamylase-type DBEs [1]. Pullulanase-type DBEs, also referred to as R-enzymes or limit-dextrinases, readily hydrolyze α(1,6) linkages in pullulan and amylopectin but have little activity toward α(1,6) linkages in glycogen. On the other hand, isoamylase-type DBEs cleave α(1,6) linkages in amylopectin and glycogen, but do not hydrolyze α(1,6) bonds in pullulan. Over the past two decades, pullulanase has been widely used in combination with α-amylase or β-amylase for the conversion of amylopectin-containing starch to glucose, maltose, and oligosaccharide, which are used in food industry [2]. Moreover, the unique catalytic activity of DBE provides a simple and effective means of producing linear short-chain glucans (SCGs) from amylopectin. SCGs have recently been reported to recrystallize in aqueous solution to form spherical nano- or micro-structures [3,4], which have emerged as a biodegradable and biocompatible carrier or encapsulation agent for various guest molecules, such as carbon nanotubes [5], iron oxide nanoparticles [6,7,8], fatty acids [9], and β-carotene [10].

The Nelson–Somogyi (NS) and 3,5-dinitrosalicylic acid (DNS) assays are the most widely used methods to measure the activity of DBEs [11]. Both methods are based on the analysis of reducing sugars generated from the enzymatic cleavage of the glycosidic bond of highly branched amylopectin or glycogen by the specific catalytic action of DBEs. In particular, the DNS assay has been intensively employed due to its simple procedure, which requires a few minutes for the reaction and spectrophotometric measurement. However, the detection limit and detection range of the DNS method is rather limited to a certain concentration, and the reaction is also affected by the degree of polymerization (DP) of carbohydrates with a reducing end group [12]. On the other hand, the NS assay is known to be 10 times more sensitive than the DNS assay, but the throughput of this method is relatively low due to the lengthy and multi-step procedure, such as heating, serial dilution and color development [13]. In addition, the partially digested amylopectin residues in a debranching reaction also contain reducing groups, which contribute to the color development in NS and DNS assays, resulting in the overestimation of the kinetic characteristics of DBEs. Thus, there is a need for a more accurate, sensitive and convenient way of quantifying the concentration of debranched glucans as well as determining the kinetic characteristics of DBEs.

Tollens’ reaction, a classical method to measure the presence of aldehyde functional groups, has also been used for a qualitative analysis of reducing sugars. The reducing sugar with an aldehyde group induces precipitation of elemental silver in a reaction containing silver nitrate and ammonia under basic conditions. That is, one aldehyde sugar molecule can be oxidized into a carboxylic acid along with the formation of electrons that can reduce silver ions to form silver nanoparticles (AgNPs). The unique plasmonic property of AgNPs generated from the Tollens’ reaction has been applied to the sensitive detection of various analytes [14,15,16,17]. Here, we employed the characteristic surface plasmon resonance of AgNPs to quantify the concentration of debranching products from amylopectin and determine the activity of DBEs. This proposed analytical system is specific to the debranching products (short-chain glucan) and exhibited up to 100 times higher sensitivity in comparison to the DNS assay. The mechanisms of this system were investigated through analyzing the AgNPs formed from the reaction, and its potential for sensitive and quantitative analysis of SCGs is presented.

## 2. Materials and Methods

### 2.1. Materials

Pullulanase, D-(+)-glucose, D-(-)-fructose, sodium potassium tartrate, sodium metabisulfite, phenol and 3,5-dinitrosalicylic acid (DNS) were purchased from Sigma-Aldrich (St. Louis, MO, USA). Silver nitrate (AgNO_3_), sodium hydroxide and ammonium hydroxide (NH_4_OH) were purchased from Daejung (Siheung, Korea). Waxy maize starch was obtained from Samyang Co (Seoul, Korea).

### 2.2. Debranching of Waxy Maize Starch

Debranching of amylopectins from waxy maize starch was carried out by the method reported by Luo et al. [18] with modifications. Briefly, waxy maize starch was dissolved in 20 mL of distilled−deionized water (DDW) to a final concentration of 0.1 mg/mL and gelatinized by microwave for 1 min. After cooling down to 60 °C, pullulanase was added to the gelatinized starch (GS) to a final concentration of 1 ASPU/mL and incubated at 60 °C for debranching. The debranched solution was immediately introduced to the modified Tollens’ reaction. ASPU is defined as the amount of enzyme that liberates 1.0 mg glucose from starch in 1 min at pH 4.4 and 60 °C.

### 2.3. Separation of SCG From the Debranching Reaction

To purify short-chain glucans (SCGs), the debranched starch (DS) solution was placed in the freezer (−20 °C) for 5 min to precipitate SCGs obtained by the debranching of amylopectin from waxy maize starch. The precipitated SCG was washed three times with DDW and stored at 4 °C until use. The molecular weight of GS and DS was measured by using high performance size exclusion chromatography (HPSEC), which consisted of a pump (model 321, Gilson, Middleton, WI, USA), an injector valve with a 200-μL sample loop (model 7725i, Rheodyne, Rohnert Park, CA, USA), a guard column (HyperGel AP, Thermo Fisher Scientific Inc., Waltham, MA, USA), a SEC column (HyperGel AP50, 7.8 mm × 300 mm, Thermo Fisher Scientific Inc.), a multi-angle laser light scattering detector (HELEOS, Wyatt Technology Corp, Santa Barbara, CA, USA), and a refractive index detector (RI-150, Thermo Electron Corp., Yokohama city, Japan). 

### 2.4. Measurement of Debranching Enzyme Activity

The debranching activity of pullulanase was measured by the modified Tollens’ reaction and DNS method. For the DNS assay, 0.1 mL of serially diluted test samples were added into 0.3 mL of a reaction solution containing 0.8% (w/v) DNS, 0.2% (w/v) phenol, 2% (w/v) sodium potassium tartrate, and 0.05% (w/v) sodium bisulfite in 1.5% (w/v) sodium hydroxide. The mixture was incubated at 100 °C for 5 min, and the absorbance of the cooled solution was measured at 540 nm using the UV-vis spectrophotometer (Infinite 200 PRO, TECAN, Zurich, Switzerland). For the modified Tollens’ reaction, 1 mL of serially diluted test samples were added into a 4 mL glass vial (Thermo Fisher Scientific, Waltham, MA, USA) containing 1 mL of reaction mixture (2 mM AgNO_3_ and 2.5 mM NH_4_OH) and incubated at 25 °C in dark incubator, in which a UV lamp (Ultraviolet Products, Inc., San Gabriel, CA, USA) was equipped to offer controlled irradiation at a distance of 10 cm away from the sample during the reaction. The absorbance of the test solution was measured at 426 nm by using the UV-vis spectrophotometer (TECAN).

### 2.5. Characterization of AgNPs Synthesized During Modified Tollens’ Reaction

The AgNPs synthesized by the modified Tollens’ reaction were washed three times with DDW and the absorption spectrum was measured by the UV-vis spectrophotometer (TECAN) at wavelengths ranging from 400 nm to 600 nm. The FT-IR absorption spectra of SCG, gelatinized starch, and AgNPs formed with SCG were analyzed using a Perkin-Elmer Spectrum One System spectrometer (Foster City, CA, USA) with KBr pellets in the range of 500 cm^−1^ to 4000 cm^−1^. For electron microscopy analysis, the AgNP suspension was dropped onto a carbon-coated copper grid (3.05 mm diameter) and dried in a desiccator under vacuum. After the sample was completely dried, the synthesized AgNPs were characterized by using field emission transmission electron microscopy (FE-TEM, JEM-2100F, JEOL, MA, USA).

## 3. Results and Discussion

### 3.1. Synthesis of AgNPs Using Debranched Starch via Modified Tollens’ Reaction

**Scheme 1:**
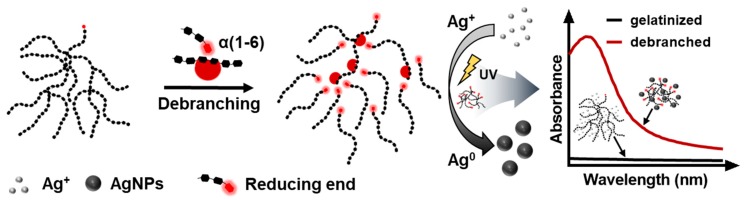
Schematic illustration showing the synthesis of silver nanoparticles (AgNPs) by short chain glucans generated from the debranching reaction, of which the synthesized AgNPs were analyzed by UV-vis spectroscopy.

Carbohydrates possessing a reducing aldehyde group are known to reduce metal ions to form metallic nanoparticles, such as silver nanoparticles (AgNPs) [19,20]. Inspired by the explicit mechanism of Tollens’ reaction and unique optical properties of AgNPs [21], we have designed a colorimetric method for the determination of the activity of starch-debranching enzyme through in situ synthesis of AgNPs (Scheme 1). 

**Figure 1:**
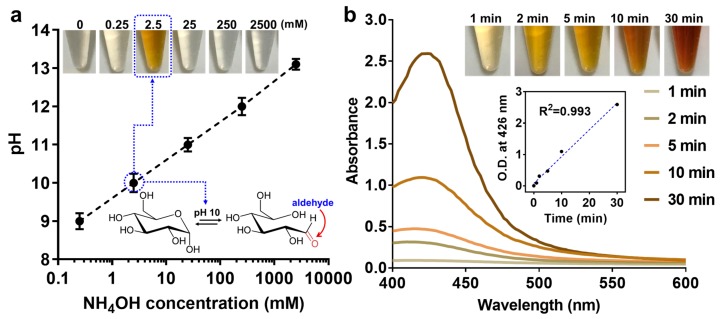
(**a**) pH of the reaction solution versus concentration of NH_4_OH. The upper inset shows the photographic image of reaction tubes containing AgNPs formed with debranched starch in the presence of varying concentrations of NH_4_OH under UV irradiation for 5 min. The inset within the plot shows the transition of glucose from a cyclic form to the open-chain aldehyde form at pH 10. (**b**) UV-vis absorption spectra of the reaction at a given time point for 30 min in the presence of debranched starch and 2.5 mM of ammonium hydroxide under UV irradiation. The debranched starch was obtained by debranching native waxy maize starch (0.1 mg/mL) using 1 ASPU/mL of pullulanase (ASPU is defined as the amount of enzyme that liberates 1.0 mg glucose from starch in 1 min at pH 4.4 and 60 °C). The upper inset shows the photographic image of the reaction tubes at each time point of the reaction. The lower inset shows the plot of the surface plasmon resonance (SPR) absorption intensity of AgNPs at 426 nm versus reaction time under UV irradiation. O. D. = optical density. R^2^ = correlation coefficient.

In this study, Tollens’ reaction was modified by combining it with UV irradiation to facilitate the rate of synthesis reaction for the formation of AgNPs [22,23]. The color transition from colorless to brown was evident in the presence of SCGs, and the intensity of absorption peak at 426 nm was proportional to the reaction time (Figure 1). The color transition reflects the reduction of silver ions to form AgNPs by oxidizing aldehyde groups in SCGs. The optimum concentration of ammonium hydroxide for the reaction was found to be 2.5 mM, which brought up the pH of reaction to 10. Since the cyclic hemiacetal group at the reducing end is in equilibrium with a ring-opened aldehyde group [24], we suggested that the free alkali (OH^−^) from NH_4_OH could act as an accelerator, inducing the oxidation of an aldehyde to carboxylic acid along with the generation of two electrons (Equation (1)) [25], which subsequently reduces the Ag+ ions to elemental silver, followed by the formation of AgNPs (Equation (2)) (Inset in Figure 1a).

(1) $$\mathrm{RCH}{\left(\mathrm{OH}\right)}_{2}+{\mathrm{OH}}^{-}\to R\left(\mathrm{CHOH}\right)O+H_{2}O+2e^{-},$$

(2) $$\mathrm{RCHO}+\;2\mathrm{Ag}{\left({\mathrm{NH}}_{3}\right)}_{2}^{+}+3{\mathrm{OH}}^{-}\to {\mathrm{RCO}}_{2}^{-}+2\mathrm{Ag}+2H_{2}O+4{\mathrm{NH}}_{3}$$

UV-vis spectra of the reaction showed distinct absorbance peaks at 410–430 nm (Figure 1b), which falls between a typical surface plasmon resonance (SPR) absorption band of AgNPs (400–450 nm) [23]. The increase in the intensity of absorbance peak as a function of UV irradiation time suggests the fact that the reducing power of SCGs to form AgNPs is enhanced significantly by UV irradiation. On the other hand, the characteristic SPR band of AgNPs was not observed from the same reaction without UV irradiation, suggesting that UV irradiation plays an important role in enhancing the rate of reaction and sensitivity of the modified Tollens’ reaction to monitor the concentration of SCGs (Figure S1).

### 3.2. Characterization of AgNPs Synthesized by Modified Tollens’ Reaction

**Figure 2:**
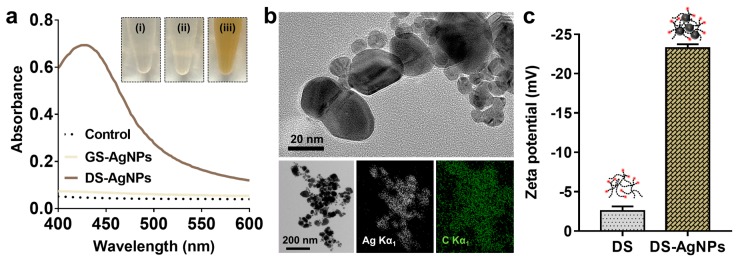
(**a**) UV-vis absorption spectra showing the absorption band of AgNPs formed without (control) and with 1 mg/mL of gelatinized starch (GS-AgNPs) or debranched short-chain glucans (SCG) (DS-AgNPs) under 5 min UV irradiation. Inset shows the photographic images of the corresponding reaction solution of control (i), GS-AgNPs (ii), and DS-AgNPs (iii). (**b**) Transmission electron microscopy (TEM) image (top) and element mapping analysis (bottom) of AgNPs formed with debranched SCG (1 mg/mL). Silver (Ag) and carbon (C) are represented in white and green, respectively. (**c**) Zeta potential of debranched starch (DS) and DS-AgNPs.

The reaction containing AgNPs formed with debranched SCGs (DS-AgNPs) showed a distinct absorbance peak at 426 nm, whereas no such a characteristic peak was observed in the same reaction carried out with gelatinized starch before debranching in equal concentration (Figure 2a). This could be attributed to a greater concentration of the reducing aldehyde end group at a given concentration of debranched SCGs when compared to that of gelatinized amylopectin, which has only one reducing end group per one amylopectin molecule. This result supports the fact that AgNPs would be formed by reducing the aldehyde end group of SCGs in a concentration-dependent manner, enabling us to determine the degree of debranching. The TEM image revealed that the AgNPs synthesized with debranched SCGs (DS-AgNPs) have a pseudo-spherical or polygonal morphology with a diameter ranging from 10 nm to 40 nm (Figure 2b). The elemental mapping analysis of DS-AgNPs exhibited the characteristic signals for silver (Ag) and carbon (C) (Figure 2b and Figure S2). The carbon signal from the DS-AgNPs would be derived from the SCGs adsorbed on the surface of AgNPs. In addition, DS-AgNPs exhibited a highly negative zeta potential (−23.2 ± 1.6 mV), while the debranched SCGs alone showed a low surface charge of −2.6 ± 0.5 mV (Figure 2c). The highly negative surface charge of DS-AgNPs is believed to be caused by the oxidation of the aldehyde group at the reducing end of the SCG to carboxylate during the reduction process of silver ions to form AgNPs. The surface-bound SCGs with a carboxyl end group would result in the increased colloidal stability of AgNPs, exhibiting a sharp and characteristic SPR spectrum of AgNPs.

**Figure 3:**
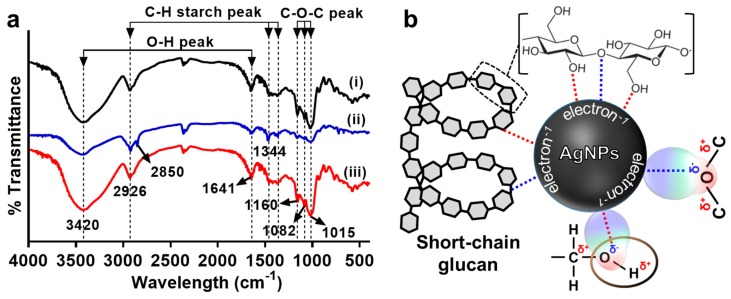
(**a**) Fourier transform infrared (FTIR) spectra of debranched SCG (i), DS-AgNPs (ii), and gelatinized starch (iii). (**b**) A cartoon illustration showing dipole–dipole interactions between AgNPs and short-chain glucans obtained from the debranching of native starch.

The interaction between debranched SCGs and AgNPs was further investigated by FTIR analysis (Figure 3a). Wavelengths in the range of 4000–3300 cm^−1^ and 1700–1600 cm^−1^ are typical absorption bands of OH stretching and carbonyl stretching, respectively. Note that the FTIR spectrum of DS-AgNPs clearly showed two bands (at 2926 cm^−1^ and 2850 cm^−1^) of C–H stretching from aldehydes in SCGs, whereas both DS and GS show a single vibrational band at 2926 cm^−1^, reflecting the presence of C–H stretching that is related to the alkyl groups [26]. This result indicates that the aldehyde group in SCGs may not be in direct contact with the surface of AgNPs in case of DS-AgNPs. It is worth noting that the stretching vibrations of OH (4000–3300 cm^−1^) and glycosidic linkage (1015–1160 cm^−1^) of DS-AgNPs were significantly decreased when compared to that of pure DS and GS, suggesting that the interaction is mainly mediated through dipole–dipole or charge–dipole interactions between the oxygen atoms in the glycosidic linkage and hydroxyl group of SCGs and the surface of AgNPs (Figure 3b).

### 3.3. Quantification of Debranched SCGs through Modified Tollens’ Reaction

**Figure 4:**
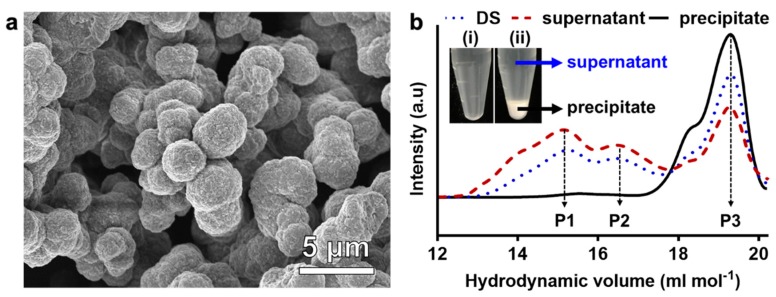
(**a**) Scanning electron microscopy (SEM) image of the white precipitate obtained by freezing the debranched starch solution at –20 °C for 5 min. (**b**) Molecular size distribution of glucan molecules after full debranching reaction (dotted line) and its supernatant (dashed line) and precipitate (solid line), measured by gel permeation chromatography (GPC). Inset shows a photographic image of the reaction tubes after debranching (i) and cooling at −20 °C for 5 min (ii). The debranching reaction was carried out with 0.1 mg/mL native waxy maize starch and 1 ASPU/mL of pullulanase for 1 h. The white precipitate is self-aggregated SCG molecules at −20 °C.

Preparation of the pure debranching products (SCGs) generated during the debranching reaction would be the first step to evaluate the accuracy and specificity of the analytical system in measuring the debranching activity of DBEs. Ethanol precipitation is effective in separating carbohydrates dissolved in water by decreasing their solubility in ethanolic solution, but selective precipitation of debranched SCG from the whole reaction is hard to achieve by this method. Here, we employed a recently developed approach to selectively separate SCGs from the debranching reaction. According to previous reports, debranched SCGs could spontaneously be assembled into a B-type crystalline microstructure, whereas partially digested amylopectins or remnants of the reaction except SCG were not incorporated into the microstructure [4,6]. Thus, we employed this principle to separate pure SCGs from the debranching reaction by using their intrinsic nature of self-assembling and precipitating at low temperature (−20 °C) for 5 min in aqueous solution. The precipitate was shown to be micro-sized spherical particles with B-type crystallinity (Figure 4a and Figure S3). The molecular weight of the precipitate was analyzed by high-pressure size exclusion chromatography (HPSEC) analysis, and the results show that the major fraction (> 99%) of the precipitate was SCGs with a main peak at 2.4 × 10^3^ g/mol (DP ≈ 13) (Figure 4b, Table S1). On the other hand, the whole debranching reaction before precipitation and the supernatant contained a larger fraction of high molecular weight glucans of 2.9 × 10^4^ g/mol (DP ≈ 160) and 8.3 × 10^4^ g/mol (DP ≈ 460) that could have originated from the partially digested amylopectins or remnants of the reaction (Table S1). These results clearly showed that precipitation of the debranched starch solution at low temperature for 5 min is a rapid and effective means of extracting high-purity SCGs. 

**Figure 5:**
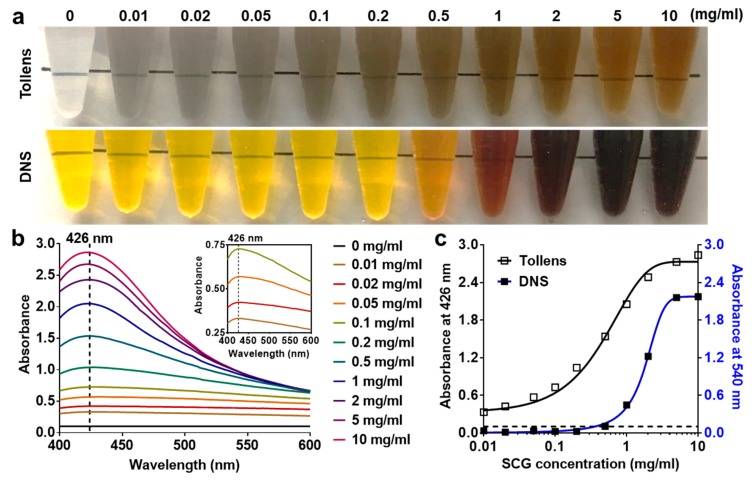
(**a**) Color development of the reaction as a function of concentration of debranched SCGs through modified Tollens’ reaction (top) and 3,5-dinitrosalicylic acid (DNS) reaction (bottom). (**b**) UV-vis absorption spectra showing the characteristic absorption peaks of AgNPs regardless of the concentration of debranched SCGs. The inset is a magnified view of the absorption spectra of AgNPs formed at the lower concentrations of SCGs. (**c**) Calibration curves for the quantitative determination of debranched SCGs through the modified Tollens’ reaction (hollow square) and DNS method (filled square). The dotted line represents the SPR absorption spectrum of AgNPs formed without SCGs (control). A curve was fitted for nonlinear regression using a sigmoidal function.

The sensitivity of this detection system for quantitative measurement of debranched SCGs was investigated in comparison to the conventional DNS method. The intensity of color development derived from the synthesized AgNPs was proportional to the concentration of SCGs ranging from 0.01–10 mg/mL (Figure 5a). The SPR absorption peaks of AgNPs generated from the modified Tollens’ reaction in the presence of varying concentration of SCGs from 0.01–10 mg/mL were all located at 426 nm, indicating that the detection limit of this detection system was 0.01 mg/mL (Figure 5b). Thus, the absorbance value at 426 nm was used for the quantitative measurement of SCGs present in the sample (Figure 5c). However, it should be noted that the characteristic SPR absorption band of AgNPs was not observed when glucose or fructose was used as a reducing agent at a concentration ranging from 0.01–10 mg/mL (Figure S4). Those monosaccharides both have an aldehyde group that is capable of reducing silver ions to form AgNPs [20], so the absence of the characteristic SPR peak might be attributed to the aggregation of the AgNPs into a micro-sized structure. Considering that glucose and fructose molecules are much smaller than SCGs, we assumed that these two monosaccharides adsorbed on the surface of nanoparticles do not act as an effective steric stabilizer to prevent the aggregation behavior of AgNPs [20]. These findings suggest that this modified Tollens’ reaction is effective for colorimetric determinations of the SCGs generated from the debranching of native starch. The calibration curve for the modified Tollens’ reaction showed a good correlation between the intensity of the absorbance peak and the amount of debranched SCGs over the concentration range of 0.01–10 mg/mL. On the other hand, notable color development by DNS assay began to take place from much higher concentration of SCGs (around 1 mg/mL). The results suggest that this detection system is specific to SCGs, not monosaccharides, and the detection limit is around 100 times lower than that of the DNS assay. 

### 3.4. Investigation of the Debranching Reaction in a Real Sample

**Figure 6:**
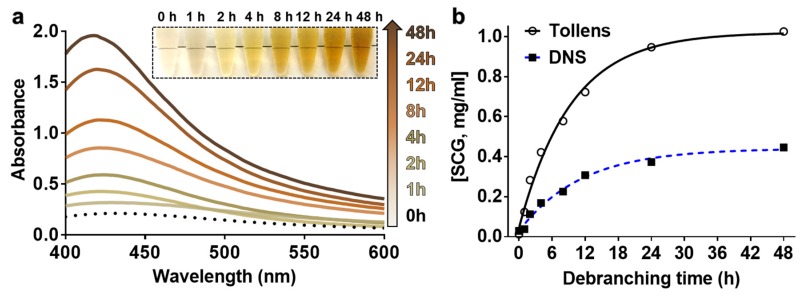
(**a**) UV-vis absorption spectra of reactions at a given time point for 48 h. The reaction was carried out with 1 mg of waxy maize starch and 1 APSU/mL of pullulanase at 37 °C. Inset shows the reaction tube at each time point. (**b**) A plot of SCG concentration versus debranching time measured by modified Tollens’ reaction (hollow round) and DNS assay (filled square). The concentration of SCG was estimated based on the calibration curve obtained by modified Tollens’ reaction and DNS assay, respectively.

The feasibility of the proposed detection system for monitoring the progress of a debranching reaction was further investigated by using a real sample. An enzymatic debranching reaction was carried out using waxy maize starch (1 mg/mL) and 1.0 APSU/mL of pullulanase, and an aliquot of the sample was taken from the reaction at a given time point over the course of 48 h reaction. Figure 6a presents the SPR absorption spectra of AgNPs formed with the sample taken from the debranching reaction at each time point. The results showed that the intensity of the SPR absorption peak at 426 nm gradually increased with the progression of the debranching reaction (Figure 6a). 

The intensity of the absorbance peak from the reaction was converted to the amount of SCG based on the calibration curves obtained from the modified Tollens’ reaction and DNS assay, respectively. According to the plot of SCGs vs. debranching time (hours), the debranching reaction reached a plateau at around 24 h from the beginning of the reaction, suggesting that all the branches in the amylopectins of waxy maize starch were hydrolyzed at the given condition. The profile of the SCGs estimated by DNS assay with the same sample showed a similar trend, but the amplitude of the signal was notably lower, particularly at the early stages of the reaction, in comparison to that of the modified Tollens’ reaction (Figure 6b). The debranching activity of pullulanase was evaluated using a first-order kinetic model [1]. The modified Tollens’ reaction showed a higher debranching rate constant (*k* = 0.237) than the DNS assay (*k* = 0.163) (Figure S5). Considering the higher sensitivity and greater fitting nature of the plot, we believe that the rate constant of the modified Tollens’ reaction would be more accurate to represent the debranching activity of DBEs. These results support that the modified Tollens’ reaction proposed in this work provides a simple, sensitive and accurate means of monitoring the activity of debranching enzyme and the progress of the reaction. 

## 4. Conclusions

In this study, we present a rapid and sensitive colorimetric assay for quantitative determination of short-chain glucans (SCGs) generated from the enzymatic debranching of amylopectin, which enabled us to monitor the debranching process and measure the activity of pullulanase-type starch-debranching enzyme. We employed the principle of Tollens’ reaction to quantify the debranching product, SCG, of which the process was significantly facilitated by UV irradiation. The SCGs with a reducing end group induced the reduction of silver ions to form AgNPs in a concentration-dependent manner, and the presence of SCGs on the surface of AgNPs increased the colloidal stability of AgNPs which was evidenced by the homogeneous color development and characteristic SPR peak. It was found that the debranched SCGs were adsorbed onto the surface of AgNPs by dipolar–dipolar and charge–dipolar interactions. Monosaccharides possessing an aldehyde group, such as glucose and fructose, could also reduce silver ions but the colloidal stability of the reduction product was too low to give a characteristic SPR absorbance band at the given reaction condition; that is, the proposed detection system is specific to the debranching product. The intrinsic nature of SCGs that precipitate in crystal form in aqueous solution at low temperature was exploited to prepare the sample that further improved the selectivity of the methods for debranched SCGs. In addition to the high selectivity, the detection limit of this method was 100 times lower than that of the conventional DNS assay, which would find its application in the sensitive and accurate measurement of debranching products and investigation of the kinetic characteristics of starch-debranching enzymes.

## Supplementary Materials

The following are available online at https://www.mdpi.com/2079-4991/9/9/1291/s1, Table S1: The ratio of small and large glucan molecules in supernatant, precipitate, and whole debranched solution (DS), Figure S1: UV-vis absorption spectra of AgNPs formed without UV irradiation for 30 min. Figure S2: EDX spectra of DS-AgNPs formed with the debranched SCGs, Figure S3: XRD analysis of white precipitate obtained by freezing the debranched starch solution at –20 °C for 5 min, Figure S4: Photographic images of reaction tubes and UV-vis absorption spectra of AgNPs formed with varying concentrations of glucose and fructose through the modified Tollens’ reaction under UV irradiation for 5 min, Figure S5: The plot of ln(*A*_0_/*A_t_*) from modified Tollens’ reaction and DNS reaction versus debranching time.
