# Supplementary material for: Colorimetric Determination of the Activity of Starch-Debranching Enzyme via Modified Tollens’ Reaction
Source: Nanomaterials (Basel). 2019 Sep 10;9(9):1291. doi: 10.3390/nano9091291 (PMC6781065; doi:10.3390/nano9091291)
Supplement: Supplementary file 1 [file nanomaterials-09-01291-s001.pdf]

nanomaterials

Colorimetric determination of the activity of starch-debranching enzyme via modified Tollens' reaction

Ke Luo, Nack-geun Kim, Sang-Mook You, and Young-Rok Kim*

Graduate School of Biotechnology & Department of Food Science and Biotechnology, College of Life Sciences, Kyung Hee University, Yongin, 17104 South Korea

* Correspondence: Tel: +82-31-201-3830; E-mail address: youngkim@khu.ac.kr

Received: date; Accepted: date; Published: date

Table S1. The ratio of small and large glucan molecules in supernatant, precipitate, and whole debranched solution (DS).

Samples	Peak 1			Peak 2			Peak 3		
	Peak area (%)	MW (g mol^{-1})	DP[a]	Peak area (%)	MW (g mol^{-1})	DP	Peak area (%)	MW (g mol^{-1})	DP
**DS**	26.74	82212	457	35.31	29619	165	37.95	2540	14
**Supernatant**	43.22	83178	462	47.37	28167	157	9.41	2409	13
**Precipitate**	ND[b]	ND	ND	ND	ND	ND	99.2	2413	13

[a] Degree of polymerization.

[b] Peak is not detected.

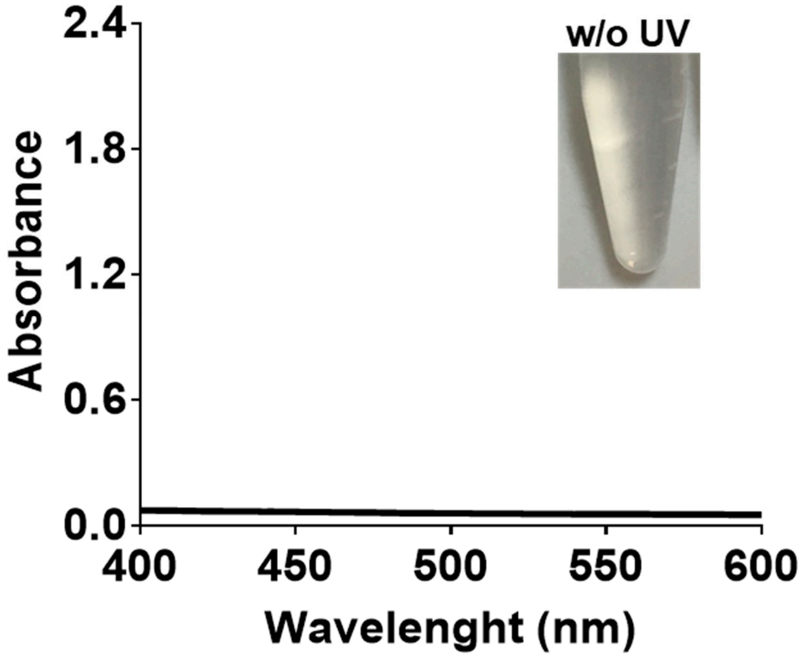

Figure S1. UV-vis absorption spectra of AgNPs formed without UV irradiation for 30 min.

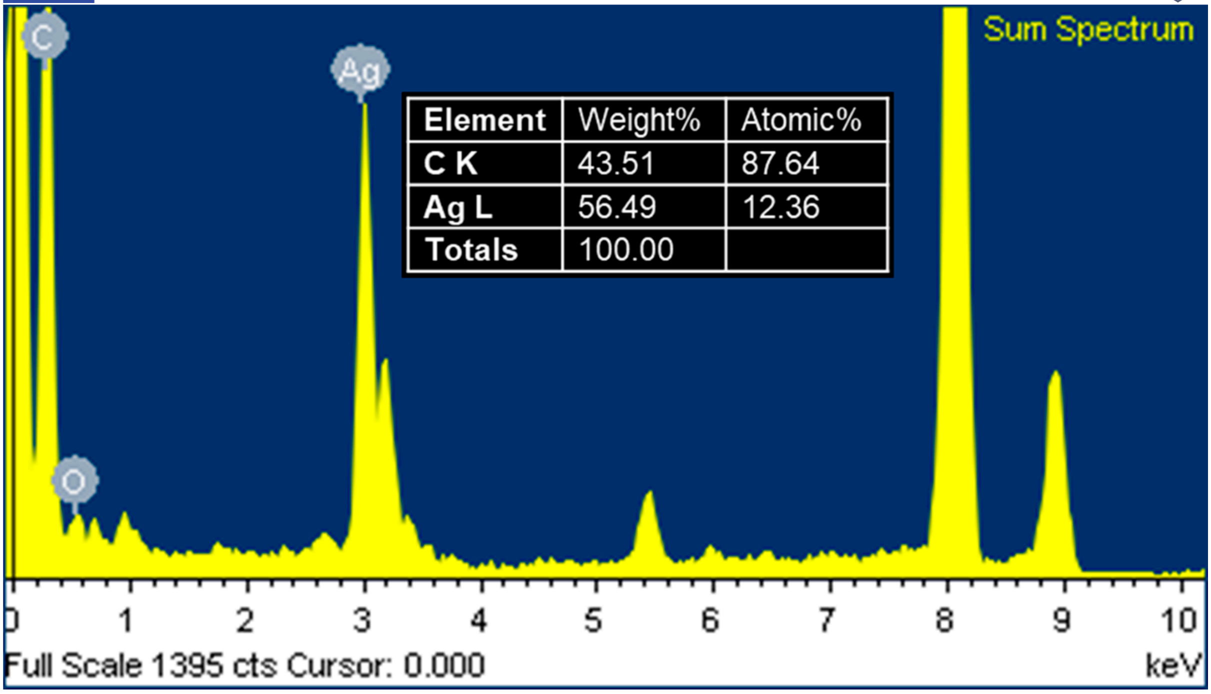

Figure S2. EDX spectra of DS-AgNPs formed with the debranched SCGs. Inset shows the weight and atomic percentages of carbon and silver in DS-AgNPs.

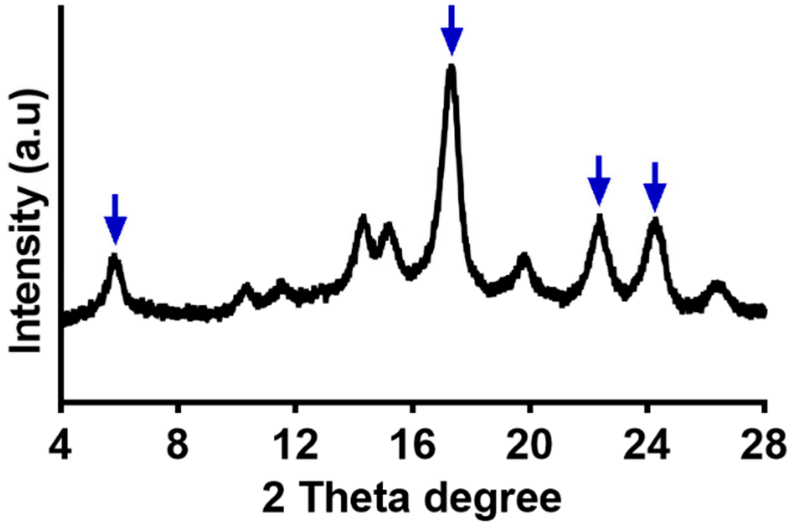

Figure S3. XRD analysis of white precipitate obtained by freezing the debranched starch solution at -20 °C for 5 min. The blue arrows represent the characteristic peaks for B-type crystal. The crystal type of SCG precipitates were analyzed from 4° to 28° (2θ) using Cu-Kα radiation on a Bruker D8 Advance X-ray diffractometer (Bruker, Karlsruhe, Germany). All samples were fully dehydrated in a vacuum desiccator before XRD analysis.

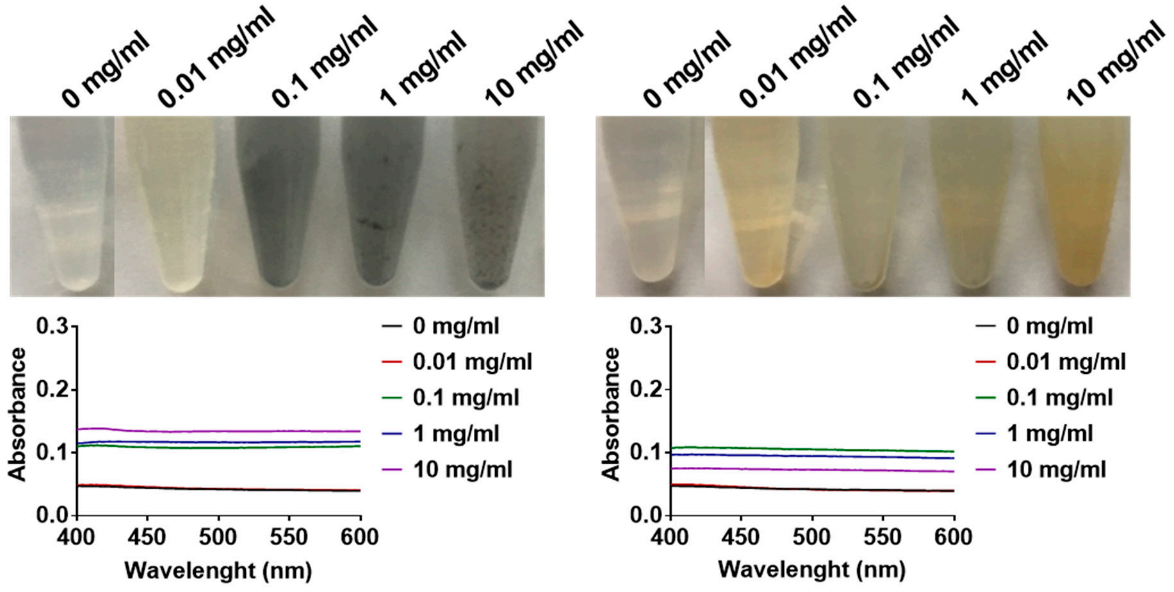

Figure S4. Photographic images of reaction tubes (up) and UV-vis absorption spectra (down) of AgNPs formed with varying concentration of glucose (left) and fructose (right) through the modified Tollens' reaction under UV irradiation for 5 min.

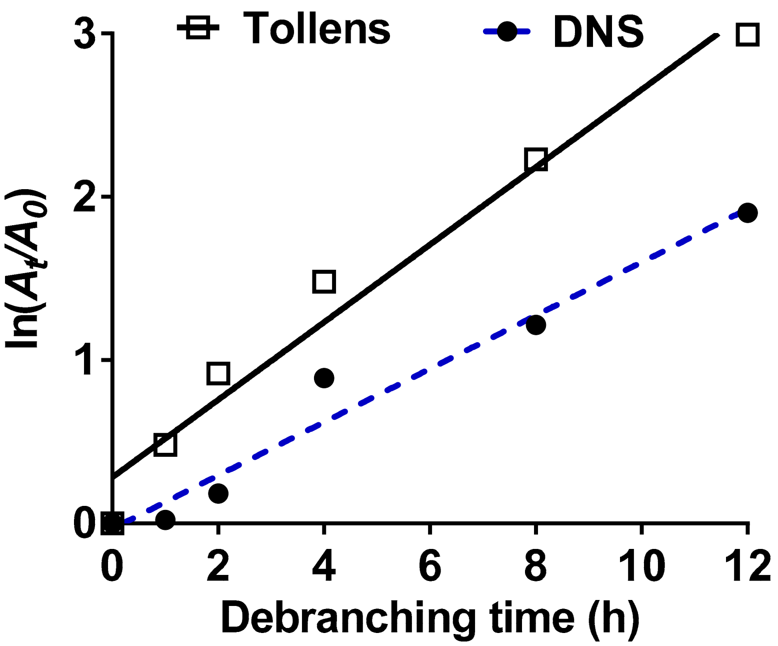

Figure S5. The plot of ln(A_0/A_t) from modified Tollens reaction (hollow square) and DNS assay (filled circle) versus debranching time.

The debranching rate constant of pullulanase was calculated by using a first-order kinetic model [1]:

$$\mathrm{Ln}(A_t/A_0) = kt \qquad (1)$$

where A_0 donates the initial absorption at 426 nm and 540 nm in the Tollens` reaction and DNS assay, respectively, and A_t is the absorption at a given time point, t, and k is the reaction rate constant.

References

1. Simonin, J.-P. On the comparison of pseudo-first order and pseudo-second order rate laws in the modeling of adsorption kinetics. *Chem. Eng. J.* **2016**, *300*, 254-263.
